# Effect of Cardamom Oil After Acute and Repeated Administration in Albino Wistar Rats

**DOI:** 10.7759/cureus.98295

**Published:** 2025-12-02

**Authors:** Sandip Auti, Yogesh A Kulkarni

**Affiliations:** 1 Shobhaben Pratapbhai Patel School of Pharmacy and Technology Management, SVKM's Narsee Monjee Institute of Management Studies (NMIMS) Deemed to be University, Mumbai, IND

**Keywords:** acute toxicity, cardamom oil, oecd, oral toxicity, repeated dose toxicity

## Abstract

Purpose*: *Cardamom is an important medicinal plant widely used in Ayurvedic medicines for therapeutic purposes. However, the toxicity study of the essential oil obtained from *Elettaria cardamomum* is not well explored. Therefore, the present study aims to design acute and repeated dose oral toxicity of cardamom oil as per the Organisation for Economic Co-operation and Development (OECD) guidelines.

Methods: A single oral dose of cardamom oil at 300 and 2000 mg/kg was administered to female Wistar rats, and they were observed for signs of behavioural changes, mortality, and morbidity for 14 days. In the case of a repeated dose toxicity study of cardamom oil, doses of 50, 100, and 200 mg/kg were administered to rats for 28 days orally. The effect of cardamom oil on body weight, food intake, consumption of water, relative organ weight, hematological and urine parameters, and clinical biochemistry profile was studied. Histopathology and gross necropsy of vital organs were performed.

Results: In an acute toxicity study, cardamom oil at a 300 mg/kg dose did not show any signs of toxicity. At a 2000 mg/kg dose, cardamom oil showed mortality in one animal, and the other two animals displayed mild signs of toxicity. Based on the results of the acute toxicity study, it can be concluded that the lethal dose (LD)₅₀ of cardamom oil is greater than 2000 mg/kg. In a repeated dose toxicity study, cardamom oil at 50, 100, and 200 mg/kg did not show any significant changes in body weight, food intake, water consumption, and relative organ weight. Cardamom oil did not show any significant changes in biochemical, renal, haematological parameters, and histopathological study as compared to the control group.

Conclusion: Cardamom oil was found to be safe at 300 mg/kg in an acute toxicity study and at all selected dose levels in a repeated dose toxicity study in rats.

## Introduction

Toxicity studies of natural products are crucial for ensuring safety and efficacy before using them in therapeutic interventions [1]. The use of medicinal plants containing essential oils is widely accepted in traditional medicine. Cardamom oil has been used in the prevention and treatment of various disease conditions and has been well documented. The chemical profile of cardamom oil is very complex, comprising various constituents at variable concentrations. Exposure to essential oils has been associated with toxicity issues in several scientific reports [2]. The Flavor and Extract Manufacturers Association (FEMA) provides 'generally recognized as safe' (GRAS) status to most of the essential oils in case of their use as flavors in food products [3,4]. Essential oils, when used at higher concentrations in various preclinical and clinical studies, could produce certain toxic effects in specific conditions [5]. While cardamom oil is generally regarded as safe in food use, essential oils can exert dose-dependent toxic effects when used in concentrated medicinal forms [4,5]. Therefore, safety evaluation of oil under controlled conditions is essential, especially when administered at pharmacological doses.

There is an unmet need to create awareness in the public domain related to the usage of medicinal plants containing essential oils [6]. Cardamom oil is obtained from the steam distillation of dried ripe fruits of *Elettaria cardamomum *(L.) (Apiaceae). It is also called the 'Queen of Spices.' It has been proven for its antiviral, gastroprotective, antioxidant, anticonvulsant, anticancer, and antidiabetic activities [7]. Cardamom oil mainly contains terpenoids such as α-terpinyl acetate (20% to 53%), 1,8-cineole (25% to 45%), linalyl acetate (8.2%), limonene (5.6%), linalool (5.4%), and other minor constituents such as β-pinene, trans-geraniol, and borneol [8]. There are limited scientific reports available related to the safety study of cardamom oil. Hence, an acute and repeated dose toxicity study of cardamom oil in rats was evaluated. 

## Materials and methods

Procedure

Cardamom oil was purchased from iFRAGRANCE INDIA (Kannauj, UP, IND). The Albino Wistar rats used in the study were procured from the National Institute of Biosciences (Pune, MH, IND) and weighed between 180 and 220 grams. They were kept in a controlled environment with a 12-hour light and dark cycle and a temperature of around 23 ± 2°C. Before the study began, the rats had a week to adjust to their surroundings. During this time, they were provided with purified water and a standard pellet diet (Nutrimix Laboratory Animal Feed; Nutrimix Feed Company, Vega Baja, PR). The protocol was approved by the Shri Vile Parle Kelavani Mandal (SVKM)'s Institutional Animal Ethics Committee (IAEC) (protocol approval no. CPCSEA/IAEC/P-70/2018).

Acute toxicity study

An acute toxicity study of cardamom oil was carried out as per the Organisation for Economic Co-operation and Development (OECD) guideline no. 423 with a slight modification [9]. The OECD 423 guideline mentions three female rats in a group for stepwise acute toxicity assessment. The animals were divided into three groups, each of which had three female rats weighing between 140 and 170 gm. Cardamom oil was suspended in 5% Tween 80 and administered in a single dose orally via gavage to animals at doses of 300 and 2000 mg/kg body weight (Table 1).

**Table 1:** Grouping for acute toxicity study

Group number	Treatment
1	5% Tween 80
2	Cardamom oil 300 mg/kg
3	Cardamom oil 2000 mg/kg

After administration of the drug, each animal was observed once within the first half hour, then at regular intervals throughout the next 24 hours, with particular focus on the first four hours, and then every day for the next 14 days [10]. Every day, any symptoms of toxicity, such as convulsions, tremors, lethargy, excessive salivation, and diarrhea, were observed [11]. The animals were closely monitored for any odd changes in their behavior and health [12]. All of the animals on day 15 of the study were humanely sacrificed. The gross necropsy of all animals was carried out, which included an examination of the contents of their cranial, abdominal, and thoracic cavities. To assess any indications of toxicity, the exterior body surface and all orifices were examined.

Repeated dose toxicity study

The OECD guideline no. 407, with slight modifications, was used for the repeated dose toxicity study of cardamom oil [13,14]. The animals were divided into four groups, with five males and five females in each group. Group I received 5% Tween 80 as a vehicle by oral route; groups II, III, and IV were administered with 50, 100, and 200 mg/kg doses of cardamom oil by using oral gavage (Table 2). The volume of the administered dose was kept at 10 mL/kg body weight for all experimental groups. The published literature served as the basis for the dose level selection. Several in vivo experiments in rats have shown oral dosage levels of cardamom oil ranging from 10 to 200 mg/kg. Doses of 10, 20, 50, 100, and 200 mg/kg of cardamom oil were administered to rodents in several scientifically documented investigations [15-17]. Therefore, in the repeated dosage toxicity study, dose levels of cardamom oil at 50, 100, and 200 mg/kg were used. The duration of dosing for all groups was given for 28 days. Every day, all animals were examined for any indications of toxicity, illness, or mortality; general clinical observations were also made.

**Table 2:** Grouping for repeated dose toxicity study

Group number	Treatment
1	5% Tween 80
2	Cardamom oil 50 mg/kg
3	Cardamom oil 100 mg/kg
4	Cardamom oil 200 mg/kg

Evaluation parameters

Every week, the experimental animals' body weight, food consumption, and water intake were examined [18]. Using an automatic hematology analyzer, blood samples were analyzed for hematological parameters at the end of the study [19]. The plasma was separated from the blood and utilized to estimate the biochemical parameters. The diagnostic test kits from Transasia Bio-Medicals Ltd. (Mumbai, MH, IND) were used. The potassium, chloride, and sodium levels were determined using the electrolyte analyzer. Metabolic cages were used to collect animal urine samples. Glomerular filtration rate and renal function tests were performed for the filtered urine samples [20]. On day 29 of the study, the animals were sacrificed, and necropsy was performed. The external surfaces of the organs, all orifices, and the contents of the thoracic, abdominal, and cranial cavities were thoroughly examined. The weights of vital organs were taken after dissection, and the relative organ weight was measured. The histopathological analysis of vital organs was performed to check for any signs of toxicity [21].

Statistical analysis 

The data on food, water intake, and body weight were analyzed by two-way analysis of variance (ANOVA) followed by a Bonferroni test. All other data were analyzed by one-way ANOVA, followed by post hoc Dunnett’s multiple comparison using GraphPad Prism 8.0 (GraphPad Software, San Diego, CA, USA). A p-value < 0.05 was considered significant. All data were expressed as mean ± standard error of mean (SEM).

## Results

Acute toxicity study

A single oral dose of cardamom oil at 300 mg/kg did not exhibit any mortality or morbidity. The behavior and appearance of animals were normal, indicating normal somatomotor function. No convulsions, salivation, diarrhea, backward walking, etc., were observed. Additionally, all animals showed normal body posture, response to handling, and sensory stimulation. During gross necropsy, no animals showed any abnormalities or pathological changes to the external surface of their organs or body cavities when given a dose of 300 mg/kg. Following three hours of cardamom oil treatment at a dose of 2000 mg/kg, two out of three animals had some abnormal clinical indications, including decreased locomotor activity and increased grooming and rearing. Abnormal motor function was observed in one animal after 26 hours. After 28 hours of dose administration, one animal was found dead (Table 3). The remaining two animals showed normal behavior and appearance after two days. All the animals showed no abnormal changes in gross necropsies.

**Table 3:** Signs of toxicity and mortality in rats in acute dose toxicity of cardamom oil ST: Sign of toxicity; NB: Normal behavior; D: Died; S: Survived. ^a^Values are expressed as an animal number.

Group	Group 1	Group 2	Group 3
Dose (mg/kg)	--	300	2000
Sign of toxicity (ST/NB)^a^	0/3	0/3	2/1
Mortality (D/S)^ a^	0/3	0/3	1/2

Repeated dose toxicity study

Body Weight, Food, and Water Intake

**Figure 1:**
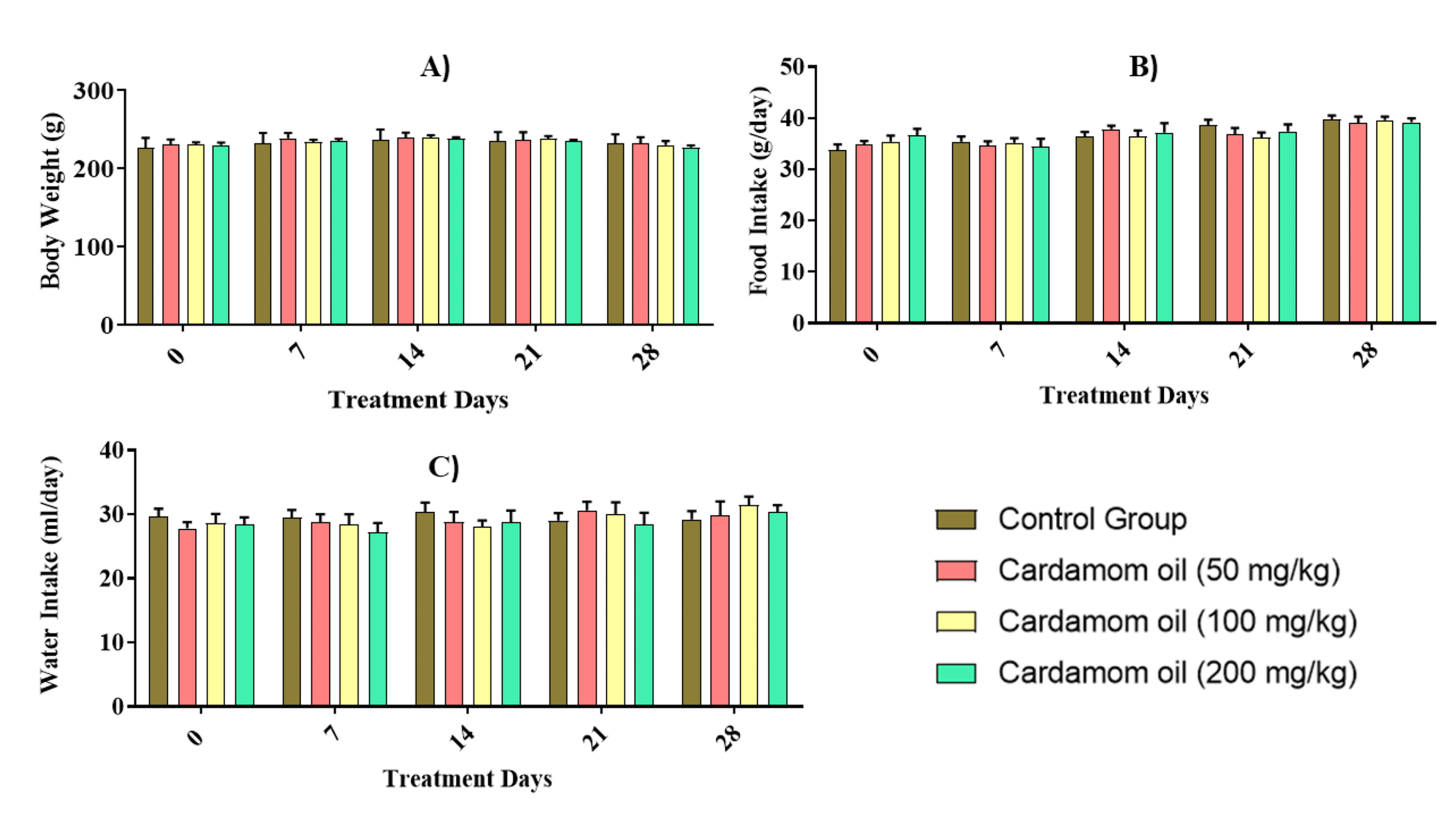
Effect of cardamom oil on body weight (A), food (B), and water intake (C) in male rats Data are expressed in mean ± SEM (n=5). The data were analyzed by two-way ANOVA followed by a Bonferroni test. The differences among the compared groups were found to be non-significant. SEM: Standard error of the mean, ANOVA: Analysis of variance

**Figure 2:**
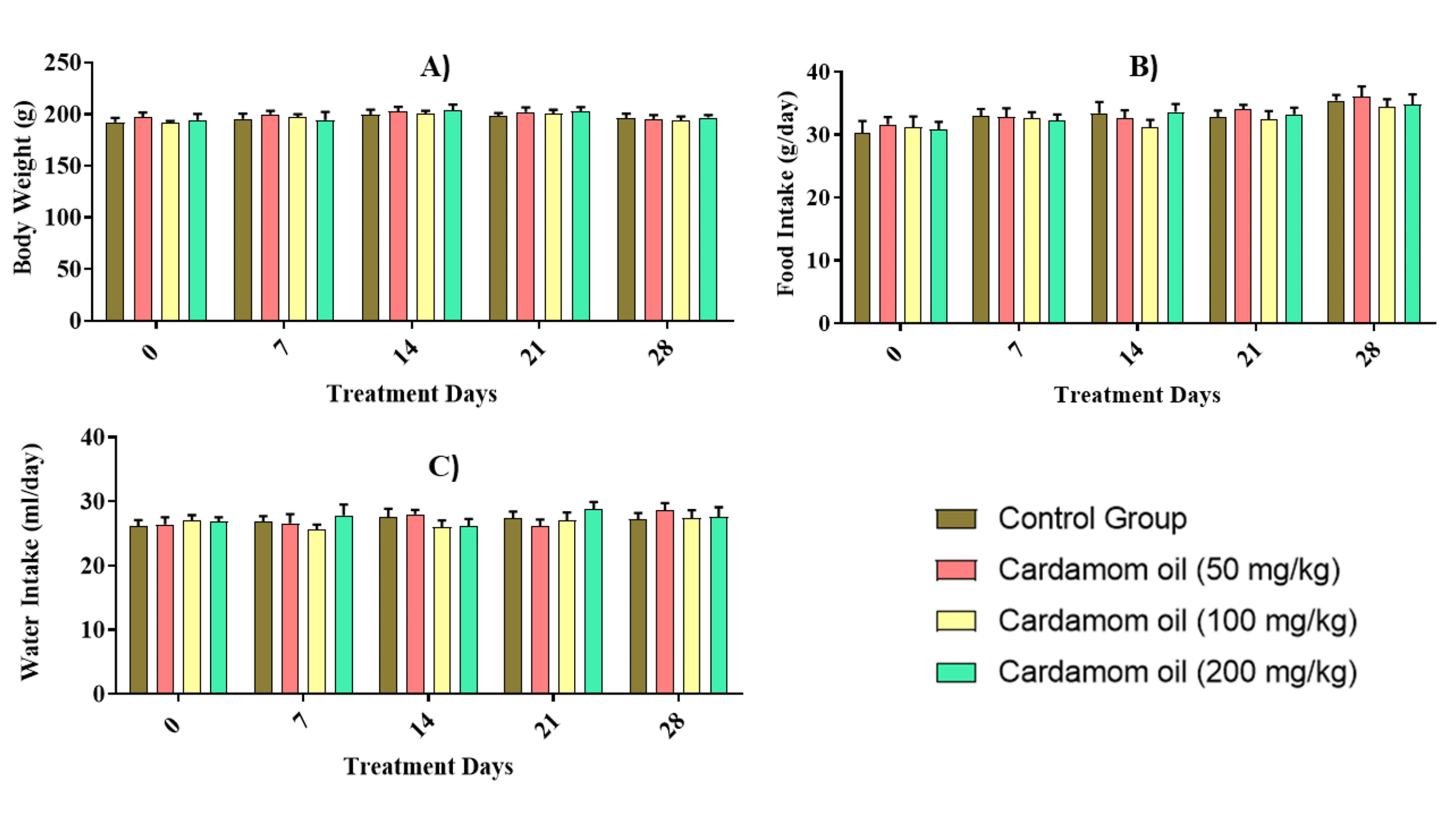
Effect of cardamom oil on body weight (A), food (B), and water intake (C) in female rats Data are expressed in mean ± SEM (n=5). The data were analyzed by two-way ANOVA followed by a Bonferroni test. The differences among the compared groups were found to be non-significant. SEM: Standard error of the mean, ANOVA: Analysis of variance

When comparing the cardamom oil-treated animals to control animals, there was no significant difference in body weight, food consumption, and water intake in both males and females (Figures 1-2).

Clinical Signs of Toxicity

Cardamom oil treatment at all selected dose levels showed no signs of toxicity in both male and female rats when compared with the control group. No change in the signs and symptoms of the central nervous system, including tremors, convulsions, sleep, coma, and lethargy, was observed in cardamom oil treatment when compared to the control group animals after 28 days.

Hematological Parameters

There were no significant changes in all hematological parameters in cardamom oil-treated groups as compared to the control group in both male and female rats (Tables 4-5).

**Table 4:** Effect of cardamom oil on hematological parameters of male rats in the repeated dose toxicity study All values are expressed as mean ± SEM (n=5). Data were analyzed by one-way ANOVA, followed by post hoc Dunnett’s multiple comparison test. The differences among the compared groups were found to be non-significant. PDW: Platelet distribution width, PCT: Plateletcrit, HCT: Hematocrit, RDW: Red blood cell distribution width, MCH: Mean corpuscular hemoglobin, RBC:  Red blood cell count, WBC: White blood cell count, HGB: Hemoglobin, MCHC: Mean corpuscular hemoglobin concentration, MPV: Mean platelet volume, MCV: Mean corpuscular volume, SEM: Standard error of the mean, ANOVA: Analysis of variance

Parameter	Control	Cardamom oil 50 mg/kg	Cardamom oil 100 mg/kg	Cardamom oil 200 mg/kg
HCT (%)	42.62±1.844	41.22±2.012	45±0.5177	43.92±0.6343
Hemoglobin (g/dl)	14.94±0.2909	14.34±0.7305	15.7±0.295	15.6±0.499
MCH (pg/cell)	19.18±0.6028	17.72±0.3277	19±0.6083	18.16±0.2839
MCHC (g/dl)	35.2±0.3536	34.88±0.1594	35.12±0.3216	34.86±0.2713
MCV (fl/cell)	53.92±0.4705	53.02±0.8605	54.24±1.522	51.8±0.6403
MPV (fl)	5.68±0.281	6.18±0.2354	5.64±0.1612	5.9±0.1789
PCT (%)	0.598±0.0538	0.568±0.0281	0.586±0.0377	0.608±0.0586
PDW (%)	15.99±0.4187	15.86±0.4654	15.4±0.1703	15.5±0.2881
Platelets (10^3^/microliter)	1002±85.89	917.4±42.11	908±61.43	905±39.45
RBC (10^6^/microliter)	7.82±0.3594	7.92±0.6099	8.428±0.115	8.57±0.2185
RDW (%)	13.44±0.1691	13.78±0.1881	13.02±0.5834	12.86±0.2249
WBC (10^3^/microliter)	18.5 ± 2.385	21.18±1.542	18.97±2.792	18.41±1.244

**Table 5:** Effect of cardamom oil on hematological parameters of female rats in the repeated dose toxicity study All values are expressed as mean ± SEM (n=5). Data were analyzed by one-way ANOVA, followed by post hoc Dunnett’s multiple comparison test. The differences among the compared groups were found to be non-significant. PDW: Platelet distribution width, PCT: Plateletcrit, HCT: Hematocrit, RDW: Red blood cell distribution width, MCH: Mean corpuscular hemoglobin, RBC:  Red blood cell count, WBC: White blood cell count, HGB: Hemoglobin, MCHC: Mean corpuscular hemoglobin concentration, MPV: Mean platelet volume, MCV: Mean corpuscular volume, SEM: Standard error of the mean, ANOVA: Analysis of variance

Parameter	Control	Cardamom oil 50 mg/kg	Cardamom oil 100 mg/kg	Cardamom oil 200 mg/kg
HCT (%)	42.92±0.5161	40.32±1.154	41.02±0.865	41.18±1.28
HGB (g/dl)	14.94±0.5221	13.64±0.186	14.44±0.4155	14.62±0.5945
MCH (pg/cell)	19.02±0.1855	18.56±0.2993	19±0.5099	18±0.2302
MCHC (g/dl)	35.22±0.5352	35.16±0.4567	35.72±0.2615	35.98±0.3382
MCV (fl/cell)	54.32±0.538	51.66±0.8352	54.31.561	51.14±1.009
MPV (fl)	5.74±0.1288	5.68±0.1068	5.48±0.2417	6.2±0.2191
PCT (%)	0.486±0.02839	0.466±0.006	0.502±0.0481	0.519±0.0315
PDW (%)	15.54±0.2293	15.84±0.24	15.2±0.3347	15.66±0.2993
Platelets (10^3^/microliter)	884±36.94	834.6±23.56	833±60.52	904.8±48.69
RBC (10^6^/microliter)	7.85±0.1897	7.476±0.1822	7.476±0.1822	7.892±0.3627
RDW (%)	12.08±0.2332	12.34±0.4202	12.02±0.28	11.98±0.2177
WBC (10^3^/microliter)	16.44±1.592	19.52±2.728	20.06±2.039	15.1±1.814

Clinical Biochemistry Parameters

Administration of cardamom oil at all selected dose levels showed no significant changes in biochemical parameters as compared to the control group (Tables 6-7). The lipid profile, electrolyte level, and liver function parameters did not show any significant changes after cardamom oil treatment in both male and female rats.

**Table 6:** Effect of cardamom oil treatment on clinical biochemistry parameters of male rats in the repeated dose toxicity study All values are expressed as mean ± SEM (n=5). Data were analyzed by one-way ANOVA, followed by post hoc Dunnett’s multiple comparison test. The differences among the compared groups were found to be non-significant. ALP: Alkaline phosphatase, ALT: Alanine aminotransferase, BUN: Blood urea nitrogen, AST: Aspartate aminotransferase, LDL: Low-density lipoproteins, HDL: High-density lipoproteins, SEM: Standard error of the mean

Parameter	Control	Cardamom oil (50 mg/kg)	Cardamom oil (100 mg/kg)	Cardamom oil (200 mg/kg)
Albumin (g/dl)	3.824±0.0551	4.03±0.1646	3.998±0.1385	4.086±0.1442
ALP (IU/L)	193±37.59	246.3±21.92	220±22.13	227.4±23.4
ALT (IU/L)	16.96±2.687	14.13±1.877	16.76±2.129	15.59±2.316
AST (IU/L)	47.15±2.161	48.72±5.391	50.85±9.246	46.85±4.966
Bilirubin (mg/dl)	0.332±0.0861	0.364±0.1064	0.334±0.06638	0.358±0.05142
BUN (mg/dl)	13.8±0.7981	14.68±0.7662	13.14±0.7271	13.04±2.181
Calcium (mg/dl)	9.534±0.1136	9.666±0.1999	9.468±0.06351	9.374±0.1207
Chloride (Mmol/L)	99.35±1.377	99.28±1.214	102.8±1.188	98.83±1.093
Cholesterol (mg/dl)	47.22±1.544	44.57±4.138	46.22±4.278	42.23±3.35
Creatinine (mg/dl)	0.764±0.1208	0.562±0.05352	0.48±0.07823	0.616±0.08078
Glucose (mg/dl)	107.5±5.137	114.3±5.626	126.7±3.526	123.5±7.989
HDL (mg/dl)	31.25±4.109	31.85±2.724	32.27±4.529	33.66±2.012
LDL (mg/dl)	48.18±5.456	45.92±4.054	47.34±4.606	44.43±2.974
Phosphorus (mg/dl)	5.564±0.3971	5.836±0.08835	5.916±0.4143	5.812±0.3054
Potassium (Mmol/L)	3.842±0.191	4.104±0.1852	3.872±0.2355	4.156±0.1831
Sodium (Mmol/L)	160±1.056	158.8±2.103	161.6±1.197	159.8±2.608
Total protein (g/dl)	6.716±0.2708	7.066±0.3897	6.698±0.4022	6.436±0.3119
Triglycerides (mg/dl)	52.64±7.854	59.54±4.762	60.67±5.407	58.25±7.883
Uric acid (mg/dl)	0.668±0.09635	0.598±0.0481	0.608±0.03942	0.68±0.09198

**Table 7:** Effect of cardamom oil treatment on clinical biochemistry parameters of female rats in the repeated dose toxicity study All values are expressed as mean ± SEM (n=5). Data were analyzed by one-way ANOVA, followed by post hoc Dunnett’s multiple comparison test. The differences among the compared groups were found to be non-significant. ALP: Alkaline phosphatase, ALT: Alanine aminotransferase, BUN: Blood urea nitrogen, AST: Aspartate aminotransferase, LDL: Low-density lipoproteins, HDL: High-density lipoproteins, SEM: Standard error of the mean

Parameter	Control	Cardamom oil (50 mg/kg)	Cardamom oil (100 mg/kg)	Cardamom oil (200 mg/kg)
Albumin (g/dl)	4.598±0.203	4.22±0.2775	4.34±0.0795	4.198±0.1136
ALP (IU/L)	172.9±9.691	179.3±12.01	185.5±7.667	183.7±4.787
ALT (IU/L)	17.45±1.444	14.66±1.501	13.69±1.826	16.31±1.303
AST (IU/L)	46.84±5.605	44.71±7.11	41.58±4.214	41.77±6.691
Bilirubin (mg/dl)	0.678±0.1694	0.612±0.1302	0.518±0.1111	0.546±0.1157
BUN (mg/dl)	14.1±0.6841	14.15±0.7082	13.66±0.5847	14.54±1.499
Calcium (mg/dl)	9.844±0.202	9.534±0.05483	9.628±0.05054	9.568±0.1612
Chloride (Mmol/L)	101±0.6627	102.3±1.119	101.4±1.739	100.7±1.742
Cholesterol (mg/dl)	46.69±4.097	50.97±3.531	45.6±3.303	44.45±4.915
Creatinine (mg/dl)	0.56±0.08444	0.484±0.05662	0.536±0.09775	0.57±0.06979
Glucose (mg/dl)	116.5±2.805	124±5.667	120.7±5.428	127.2±11.61
HDL (mg/dl)	29.06±3.912	30.84±3.812	30.4±1.861	32.94±1.856
LDL (mg/dl)	48.24±5.326	49.21±6.446	48.05±4.426	44.3±3.007
Phosphorus (mg/dl)	4.894±0.3125	4.944±0.2793	4.906±0.1991	4.808±0.1985
Potassium (Mmol/L)	3.804±0.08571	4.066±0.07743	3.66±0.1964	3.76±0.2702
Sodium (Mmol/L)	159.9±0.5334	161.1±0.804	161.6±1.746	160.8±1.789
Total protein (g/dl)	7.098±0.3933	7.174±0.3404	6.698±0.5089	6.888±0.3023
Triglycerides (mg/dl)	77.72±0.9407	64.03±9.607	67.01±8.864	72.66±10.51
Uric acid (mg/dl)	0.653±0.1309	0.714±0.04697	0.696±0.04643	0.644±0.06392

Renal Function Parameters

When compared to the control group, the renal function parameters were not significantly altered after 28 days of cardamom oil treatment at any of the selected dose levels (Tables 8-9).

**Table 8:** Effect of cardamom oil on renal function parameters of male rats in the repeated dose toxicity study All values are expressed as mean ± SEM (n=5). Data were analyzed by one-way ANOVA, followed by post hoc Dunnett’s multiple comparison test. The differences among the compared groups were found to be non-significant. SEM: Standard error of the mean, ANOVA: Analysis of variance

Parameter	Control	Cardamom oil 50 mg/kg	Cardamom oil 100 mg/kg	Cardamom oil 200 mg/kg
Albumin excretion (mg/24 h)	4.662±0.6449	5.372±0.866	3.938±0.678	6.114±1.43
Creatinine clearance (mg/ml/min)	0.54±0.08792	0.628±0.032	0.788±0.149	0.696±0.143
Glomerular filtration rate (ml/min)	0.816±0.1833	0.904±0.079	0.914±0.090	1.116±0.229
Protein excretion (mg/24h)	31.11±5.03	33.78±4.138	28.31±4.515	33.07±6.366
Urea clearance (mg/ml/min)	1.098±0.3025	1.186±0.177	1.042±0.129	1.534±0.322
Urine output (ml/24h)	4.74±0.4946	4.62±0.2818	4.28±0.4164	4.8±0.345

**Table 9:** Effect of cardamom oil on renal function parameters of female rats in the repeated dose toxicity study All values are expressed as mean ± SEM (n=5). Data were analyzed by one-way ANOVA, followed by post hoc Dunnett’s multiple comparison test. The differences among the compared groups were found to be non-significant. SEM: Standard error of the mean, ANOVA: Analysis of variance

Parameter	Control	Cardamom oil 50 mg/kg	Cardamom oil 100 mg/kg	Cardamom oil 200 mg/kg
Albumin excretion (mg/24h)	5.144±1.124	6.87±1.057	5.81±0.5814	6.366±1.237
Creatinine clearance (ml/min)	0.652±0.139	0.718±0.074	0.836±0.283	0.586±0.054
Glomerular filtration rate (ml/min)	0.896±0.1211	1.042±0.064	1.082±0.176	0.946±0.084
Protein excretion (mg/24h)	30.34±7.351	37.53±5.181	37.34±6.651	35.6±3.289
Urea clearance (ml/min)	1.142±0.1374	1.364±0.076	1.328±0.153	1.304±0.170
Urine output (ml/24h)	4.3±0.4669	4.96±0.2159	4.98±0.38	4.88±0.2354

Gross Necropsy, Relative Organ Weight, and Histopathology

The gross necropsy showed no alterations in the morphology of vital organs at any of the selected dose levels of cardamom oil treatment. When compared to control group animals, cardamom oil at doses of 50 mg/kg, 100 mg/kg, and 200 mg/kg did not significantly alter the relative organ weights of male and female rats (Tables 10-11). Histopathological studies of vital organs showed no signs of toxicity in all groups.

**Table 10:** Effect of cardamom oil on percent relative organ weight of male rats in the repeated dose toxicity study All values are expressed as mean ± SEM (n=5). Data were analyzed by one-way ANOVA, followed by post hoc Dunnett’s multiple comparison test. The differences among the compared groups were found to be non-significant. SEM: Standard error of the mean, ANOVA: Analysis of variance

Organ	Control	Cardamom oil 50 mg/kg	Cardamom oil 100 mg/kg	Cardamom oil 200 mg/kg
Adrenal gland	0.036±0.003536	0.0354±0.004697	0.0392±0.002088	0.0376±0.002874
Brain	0.97±0.06993	1±0.02588	1.026±0.06838	1.024±0.0314
Heart	0.459±0.02585	0.4706±0.01935	0.4778±0.0233	0.498±0.01619
Kidney	0.4696±0.03097	0.4618±0.01503	0.4636±0.01795	0.4608±0.01135
Liver	4.624±0.2027	4.544±0.1847	4.715±0.281	4.824±0.1823
Lung	1.052±0.07242	1.054±0.03265	1.06±0.02302	1.132±0.04465
Spleen	0.6236±0.02769	0.5934±0.02271	0.6684±0.02471	0.612±0.02376
Stomach	0.874±0.02293	0.862±0.05625	0.892±0.04306	0.932±0.02131
Testes	0.5744±0.0144	0.5752±0.0217	0.5926±0.02401	0.5816±0.01852

**Table 11:** Effect of cardamom oil on percent relative organ weight of female rats in the repeated dose toxicity study All values are expressed as mean ± SEM (n=5). Data were analyzed by one-way ANOVA, followed by post hoc Dunnett’s multiple comparison test. The differences among the compared groups were found to be non-significant. SEM: Standard error of the mean, ANOVA: Analysis of variance

Organ	Control	Cardamom oil 50 mg/kg	Cardamom oil 100 mg/kg	Cardamom oil 200 mg/kg
Adrenal gland	0.0282±0.003292	0.0294±0.001364	0.0326±0.00328	0.0304±0.002182
Brain	0.926±0.04986	0.85±0.04087	0.876±0.02379	0.868±0.0812
Heart	0.4302±0.02682	0.4124±0.02471	0.3952±0.01878	0.4098±0.01723
Kidney	0.3932±0.02956	0.3868±0.02624	0.388±0.008556	0.3832±0.02116
Liver	3.908±0.1812	3.696±0.3326	3.835±0.2121	3.835±0.1413
Lung	0.858±0.0616	0.854±0.02943	0.854±0.0413	0.86±0.02983
Ovary	0.032±0.001817	0.0326±0.001749	0.0346±0.001965	0.034±0.003178
Spleen	0.5592±0.03989	0.5252±0.02456	0.5232±0.01636	0.5254±0.02347
Stomach	0.684±0.05134	0.69±0.05683	0.762±0.02922	0.788±0.02245

## Discussion

Acute toxicity studies are typically conducted on laboratory animals at high doses (sufficient to cause mortality) [9]. Animals that regularly consume food and water have healthy physiological processes and normal nutrient intake. Alterations in food and water consumption in animals, as well as changes in body weight, suggest that the treatment may have a harmful effect [22]. Since there were no prior scientific studies on cardamom oil toxicity, we started with a 300 mg/kg dose. During the observation period, no toxicity symptoms were observed, and there were no significant variations in body weight or food and water intake at 300 mg/kg, confirming that cardamom oil is well tolerated at a 300 mg/kg dose. One rat showed mortality after receiving 2000 mg/kg of cardamom oil due to abnormal motor functions and convulsions, while the other two animals showed temporary mild toxicity signs such as rearing, grooming, and mild lethargy. Based on the results of the acute toxicity study, it can be concluded that the lethal dose (LD)₅₀ of cardamom oil is greater than 2000 mg/kg. The repeated dose toxicity study showed that cardamom oil was safe at all selected dose levels because there was no change in body weight, food intake, and water consumption after 28 days of treatment.

The adverse effects of chemicals and drugs are specified by changes in body weight. Changes in organ weight can be a sensitive sign of organ toxicity caused by chemicals, such as organ hypertrophy [23]. Therefore, determining the relative organ weight is a vital parameter in toxicity studies. There was no significant change in the relative organ weight of the cardamom oil treatment group with the control group animals at selected dose levels.

The hematopoietic system, a very sensitive and significant indicator of pathological conditions in both humans and animals, is mainly affected by hazardous substances [19]. After being exposed to toxicants, the change in hematological parameters such as platelets, RBCs, WBCs, etc., indicates disease conditions such as thrombocytopenia, anemia, etc. [24]. According to the results, cardamom oil did not interfere with the processes of hematopoiesis. When compared to the control group, no significant changes were observed in hematological parameters such as hematocrit, hemoglobin, plateletcrit (PCT), mean corpuscular hemoglobin (MCH), mean corpuscular volume (MCV), and mean corpuscular hemoglobin concentration (MCHC) following a 28-day cardamom oil treatment. These results indicate that there is no hematotoxic effect at any of the dose levels of cardamom oil.

Toxicity and pathological states are monitored to evaluate specific organ toxicity. The evaluation of cardiac, renal, and hepatic function tests is assessed as fingerprint indicators of toxicity [25]. The renal toxicity is monitored by evaluating the urine parameters such as urine output, glucose, proteins, and waste products like creatinine, urea, blood urea nitrogen (BUN), and glomerular filtration rate [20,26]. Cirrhosis, fibrosis, and liver damage are the indicators of hepatotoxicity [27]. Biochemical parameters such as triglycerides, cholesterol, low-density lipoprotein (LDL), high-density lipoprotein (HDL), total protein, glucose, creatinine, bilirubin, and uric acid did not significantly change after cardamom oil administration, which indicates no harmful effects on the liver and kidney.

Electrolytes are essential for preserving the body's equilibrium of homeostasis. Sodium keeps the body's acid-base balance stable [28]. Pathological situations occur due to changes in the electrolyte balance of plasma. When compared to the control group, treatment with cardamom oil did not significantly alter plasma electrolyte (sodium, potassium, chloride) levels, suggesting that electrolyte levels are not adversely affected after 28 days of cardamom oil treatment. Humans and animals, on exposure to a variety of hazardous substances, experience an increase in oxidative DNA damage, which leads to tissue necrosis [29]. Histopathological analysis is used in the evaluation of organ damage at the cellular level caused by the harmful effects of chemicals. The cardamom oil-treated animals showed no significant change in the histology of vital organs as compared to the control group. The findings of the biochemical and renal function tests support the safety profile of cardamom oil in rats.

In a previously published study, cardamom oil was administered intraperitoneally to mice at dose levels of 0.25, 0.5, 0.75, 1, and 1.5 mL/kg and did not cause any toxic symptoms or animal death until a dose of 0.75 mL/kg. However, the mice exhibited signs of toxicity, including drowsiness and staggering gait, at a dose of 1 mL/kg, and had 50% mortality [30]. In the present study, the cardamom oil was administered by the oral route, and it was found safe up to 200 mg/kg after repeated administration for 28 days. The present study was performed on albino Wistar rats for only 28 days, which limits the detection of long-term toxic effects of cardamom oil in animals. The species-specific differences with respect to metabolism and physiology of animals may alter the toxicological interpretations. The main limitation of the study is that we could not perform carcinogenicity, mutagenicity, genotoxicity, or reproductive and developmental toxicity tests on cardamom oil. In the future, carcinogenicity, mutagenicity, genotoxicity, and reproductive and developmental toxicity of cardamom oil need to be studied.

## Conclusions

In this acute toxicity study, no significant variations in body weight or food and water intake were observed after administration of a 300 mg/kg dose of cardamom oil. Therefore, cardamom oil was found to be safe at a 300 mg/kg dose. In the repeated dose toxicity study, cardamom oil did not show any significant changes in hematological, biochemical, or renal parameters. Also, it did not show any toxicity signs in vital organs. Cardamom oil was found to be safe at 50, 100, and 200 mg/kg doses in repeated dose toxicity. The no observed adverse effect level (NOAEL) for cardamom oil was established at 200 mg/kg/day for repeated oral administration for 28 days in Wistar rats.
